# Research on the Key Technology of a Fluorescence Detection Device Using the RT-LAMP Method for Instant Detection

**DOI:** 10.3390/mi15081044

**Published:** 2024-08-18

**Authors:** Hongzhuang Guo, Ping Gong, Tingting Sun, Xin Wang, Hao Zhang

**Affiliations:** 1 School of Physics, Changchun University of Science and Technology, Changchun 130022, China; guohongzhuang5221@163.com (H.G.); suntingting940113@163.com (T.S.); 2 Changchun Gitech Biotechnology Co., Changchun 130022, China; 3 School of Life Science and Technology, Changchun University of Science and Technology, Changchun 130022, China; gp@cust.edu.cn

**Keywords:** coronavirus tests, COMSOL simulation, Zemax simulation, optical signal detection, POCT

## Abstract

As of 31 October 2023, there have been 771,795,258 confirmed cases of COVID-19 globally. Developing simple, portable, and reliable testing devices has become increasingly important. This paper presents a point-of-care testing (POCT) device for COVID-19 based on the dual-excitation fluorescence RT-LAMP method, which is derived from the principles of RT-LAMP-based COVID-19 detection kits available in the market. The key design solutions of the device were simulated and modeled. Key performance metrics such as detection repeatability and linearity were validated. Comparative experiments with the RT-qPCR detection method were conducted to verify the accuracy and reliability of the device. Additionally, the device’s detection sensitivity and accuracy were assessed. Experimental results show that the repeatability coefficient of variation (CV) value is ≤0.09%; the linearity R^2^ for the FAM channel is 0.9977 and that for the HEX channel is 0.9899; it exhibits good anti-interference performance, with negligible cross-channel interference; the temperature stability is ±0.062 °C, the temperature accuracy is less than 0.2 °C, and there is no significant temperature overshoot during the heating process. Compared with the real-time quantitative PCR (RT-qPCR) instrument, the positive agreement rate is 100% and the negative agreement rate is 95.0%. This research provides a foundational basis for the development of equipment for the prevention of infectious diseases and clinical diagnostics.

## 1. Introduction

As of 31 October 2023, WHO pandemic reports indicate that there has been a total of 771,795,258 confirmed cases of COVID-19 globally, with a cumulative death toll of 6,977,983 [1]. The development of simple, portable, and accurate diagnostic devices has become particularly crucial.

Yongqiang Cheng et al. developed a biochemical analyzer integrating laser-induced fluorescence with a microfluidic chip [2]. This instrument exhibits high sensitivity but requires specific microfluidic chips as consumables. Mengyuan Xie et al. proposed a portable quantitative fluorescence immunoassay analyzer [3]. This device is characterized by its simple structure and low cost but is limited to a single excitation wavelength, making it unsuitable for projects that require the detection of internal reference genes. Ji M. and colleagues combined centrifugal technology with microfluidic technology to create an RT-PCR system capable of detecting influenza viruses within 1.5 h [4]. Notomi T. et al. developed a POCT (point-of-care testing) LAMP (loop-mediated isothermal amplification) detection technique, which has been widely adopted [5]. Lin, Pei-Heng and Mahardika, Ignasia Handipta et al. developed a point-of-care testing (POCT) nucleic acid detection device based on the loop-mediated isothermal amplification (LAMP) method using a microfluidic chip, which demonstrated high sensitivity and accuracy. Additionally, the creation of a LAMP-based POCT-type nucleic acid detection device for the identification of nucleic acids was outlined in the paper [6,7]. In the article “Mini review: recent progress in RT-LAMP enabled COVID-19 detection”, Thompson, Dorian et al. present a concise overview of the principles and recent developments in the use of LAMP for the detection of COVID-19 [8]. Natsuhara D. et al. utilized microfluidic chip structures for fluid distribution and employed colorimetric principles to achieve COVID-19 detection [9]. De Oliveira K.G. et al. designed a device based on the LAMP method for COVID-19 detection [10]. This device uses a gyroscope-controlled centrifugal microfluidic system, offering convenient operation but with relatively poor accuracy.

The most common methods for detecting SARS-CoV-2 include the enzyme-linked immunosorbent assay (ELISA), chemiluminescent immunoassay, real-time quantitative polymerase chain reaction (RT-qPCR), and loop-mediated isothermal amplification (RT-LAMP) [11,12,13]. Among these, RT-qPCR is considered the “gold standard” for SARS-CoV-2 detection due to its high sensitivity and specificity [14]. This study focuses on the design of a point-of-care testing (POCT) device for COVID-19 detection based on the RT-LAMP method [15,16], including simulations and modeling of key design elements. The reproducibility and linearity of the device’s detection capabilities were validated. Additionally, comparative experiments with the RT-qPCR method were conducted to verify the device’s accuracy and reliability. This study also confirmed the device’s detection sensitivity and accuracy.

## 2. Materials and Methods

### 2.1. Reagents and Instruments

The tools, apparatus, and reagents required for the experiment are shown in Table 1.

### 2.2. Principles and Programs

#### 2.2.1. Design Principle

Bst 2.0 DNA polymerase, derived from Bacillus stearothermophilus DNA Polymerase I, exhibits 5′→3′ DNA polymerase activity and strong strand displacement capability, but lacks both 5′→3′ and 3′→5′ exonuclease activities. Compared to the wild-type Bst DNA polymerase large fragment, Bst 2.0 DNA polymerase significantly enhances amplification speed and yield. Bst 2.0 HS, a hot-start isothermal polymerase, is obtained by reversibly modifying Bst 2.0 DNA polymerase. This modification completely inhibits enzyme activity at room temperature, allowing reactions to be set up at room temperature without non-specific amplification, thus improving reaction efficiency.

For the SARS-CoV-2 target sequence, six specific LAMP primers were designed. Using Bst 2.0 HS isothermal polymerase (Biori, Zhuhai, China) and the specific primers, six independent regions of the target sequence are recognized and amplified isothermally, producing double-stranded DNA that binds to Eva Green. Upon excitation at a specific wavelength, this complex emits a fluorescent signal, and the intensity of this signal is measured to determine the amount of the target substance [17].

**Figure 1:**
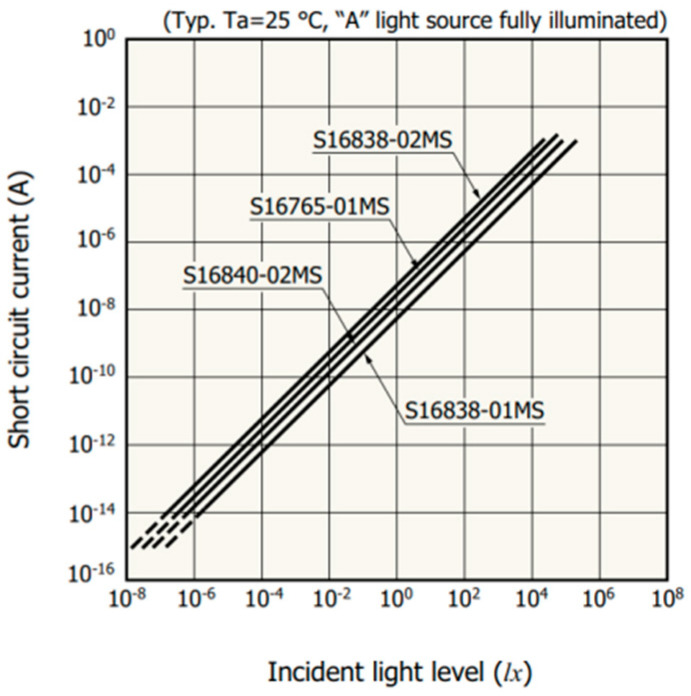
Photocurrent versus irradiance plot. In this system of coordinates, the horizontal axis represents irradiance, while the vertical axis represents light current intensity. It can be demonstrated that the light current intensity is proportional to the logarithm of the light irradiance. Consequently, the light irradiance can be calculated from the light current intensity.

The design employs the S16840-02MS silicon photodiode, manufactured by Hamamatsu in Japan, as the photoelectric sensor. When light shines on the sensor’s detection window, the sensor generates a photocurrent that has a logarithmic relationship with the light intensity [18,19]. The relationship between the photocurrent and irradiance intensity can be obtained from the manufacturer’s datasheet, as illustrated in Figure 1, with the mathematical expression provided in Equation (1).

(1) $$A=K_{1}\times lg(E)+b_{1}$$

In the equation, A is the photocurrent, K_1_ is the slope (constant), E is the irradiance, and b_1_ is the intercept.

Given that the substance concentration is directly proportional to the light intensity, we can derive Equation (2):

(2) $$C=K_{2}\times E$$

where C is the concentration of the substance, K_2_ is the proportionality constant, and E is the irradiance.

This relationship allows us to connect the photocurrent generated by the photodiode to the concentration of the target substance through the irradiance.

#### 2.2.2. Overall Design of the Device

To enhance the versatility of the device, two excitation light sources are employed: FAM LED (475 nm) and HEX LED (525 nm). The HEX laser light, after being focused by a lens, passes through a dichroic mirror and then through a small aperture beneath the incubation chamber to illuminate the bottom of the sample tube. The fluorescence emitted by the fluorescent markers in the sample, once excited, passes through a small aperture on the left side of the incubation chamber, then through an optical filter, and finally reaches the photodiode detection window.

**Figure 2:**
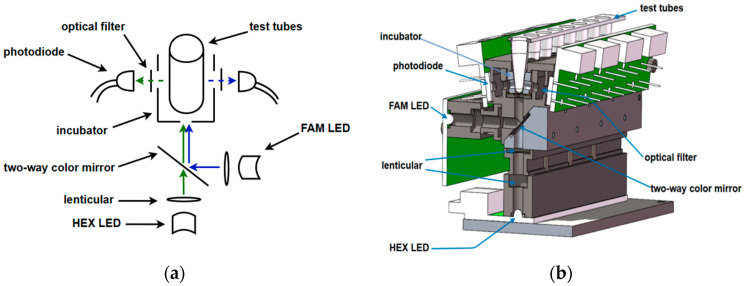
Overall design of the device: (**a**) schematic diagram of the overall design of the device; (**b**) 3D drawing of the overall design of the installation.

For the FAM excitation light, it is focused by a lens and reflected by the dichroic mirror before passing through a small aperture beneath the incubation chamber to illuminate the bottom of the sample tube. The excited fluorescence travels through a small aperture on the right side of the incubation chamber, then through an optical filter, and finally reaches the photodiode detection window on the right side. The overall design schematic of the device is shown in Figure 2a, and the 3D design is shown in Figure 2b. (Additionally, changes in light intensity due to the turbidity and color variation in the test sample can also be detected by the photodiodes).

**Figure 3:**
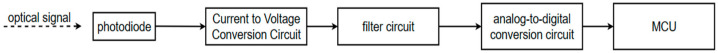
Light intensity detection block diagram.

When the emitted light strikes the detection window of the photodiode, the photodiode generates a reverse photocurrent. This current is converted to a voltage signal by a current-to-voltage conversion circuit. The voltage signal is then processed through filtering and amplification circuits. Subsequently, an analog-to-digital conversion circuit converts the voltage signal into a digital signal, which is sent to the microcontroller (STM32F103ZET6, STMicroelectronics, Geneva, Switzerland) for computation to determine the concentration of the target substance. The block diagram of the light intensity detection system is shown in Figure 3.

#### 2.2.3. Temperature Control Design

**Figure 4:**
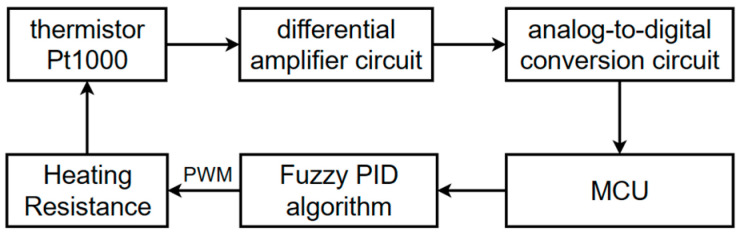
Temperature control block diagram.

The device uses a Pt1000 thermistor (CHUANJU ELECTRONICS, Guangzhou, China) as the temperature sensor. The voltage value across the thermistor is detected using a differential amplification circuit. This voltage signal is then converted to a digital signal by an analog-to-digital conversion circuit and sent to the MCU. A fuzzy PID algorithm is employed to adjust the duty cycle of the PWM (Pulse Width Modulation) signal, which controls the power of the heating resistor, thereby achieving temperature control. The temperature control block diagram is shown in Figure 4.

## 3. Results and Discussion

### 3.1. Simulation Analysis

#### 3.1.1. Incubator Temperature Distribution and Thermal Deformation 

##### COMSOL Simulation

The incubation chamber was designed using SolidWorks(R) Premium 2021 software, as shown in Figure 2b. The incubation chamber and test tube models were then imported into COMSOL Multiphysics 5.4 software. Probes were added at the temperature sensor position on the left side of the incubation chamber (incubator probes) and at the contact point between the bottom side of the inner wall of the test tube and the incubation chamber (test tube probes).

The simulation conditions were set as follows: the incubation chamber material is Aluminum 6061, and the test tube material is acrylic plastic. The initial temperature is set to 25 °C, with a generalized inward heat flux of 15 W/m^2^. A constant heat source is applied to the bottom surface of the incubation chamber.

**Figure 5:**
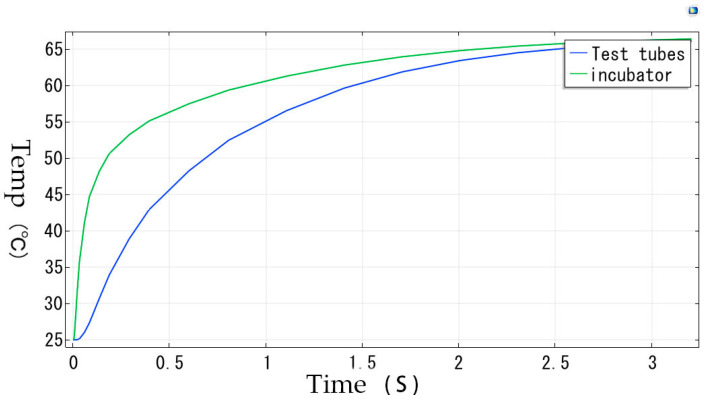
Temperature rate graph.

The simulation results indicate that the temperature at the contact point between the bottom of the test tube and the incubation chamber, as well as the overall temperature uniformity within the incubation chamber, is well-maintained. The temperature stabilizes at 65 °C. The apparatus is capable of maintaining the requisite temperature for the incubation process. The results of the simulation are presented in Figure 5.

In COMSOL software, a solid mechanics physics field was added using the default settings. A 3D plot group was then created in the results section, and a surface plot was added to this group. The expression was set to display the total displacement under the solid mechanics physics field to observe thermal expansion deformation under heating conditions.

The simulation results show that the maximum thermal expansion displacement is 2.0 × 10^−11^ mm. This minute displacement can be considered negligible, indicating that thermal deformation does not affect optical detection.

#### 3.1.2. Excitation Light Path Zemax Simulation

**Figure 6:**
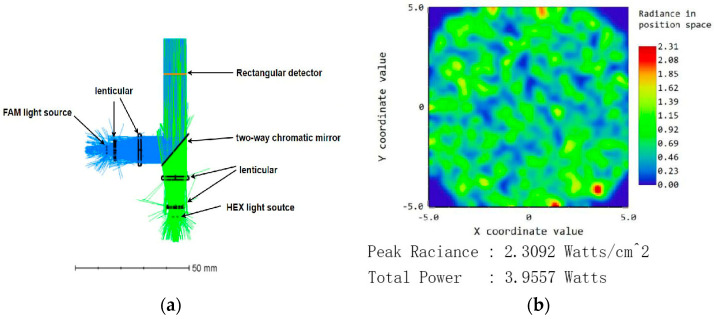
Dual-light simulation diagram: (**a**) dual optical path simulation diagram; (**b**) detector receiving diagram.

In ZEMAX OpticStudio 19.4 software, the FAM and HEX light sources were set to 3 W each, and the analysis was conducted with 1000 rays. The detector received a total light power of 3.96 W, resulting in a light source efficiency of 66.0%. The results are illustrated in Figure 6a,b.

When analyzing the HEX light source individually, the analysis fiber for the FAM light source was set to 0. The total power received by the detector was 2.02 W, with a light source efficiency of 67.3%. Conversely, when analyzing the FAM light source individually, the analysis fiber for the HEX light source was set to 0. The total power received by the detector was 2.03 W, with a light source efficiency of 67.7%.

**Figure 7:**
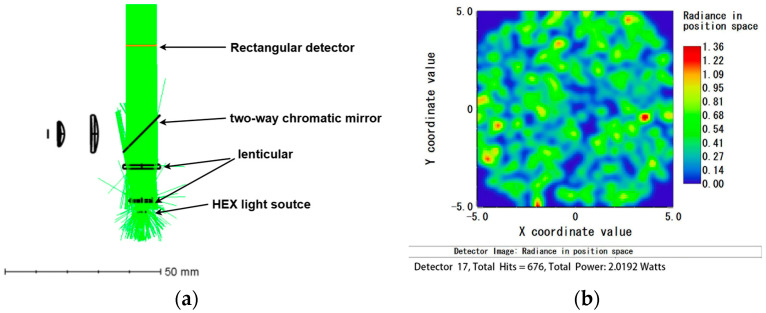
HEX light simulation diagram: (**a**) optical path simulation when HEX light is working alone; (**b**) detector receiving diagram when HEX light is working alone.

**Figure 8:**
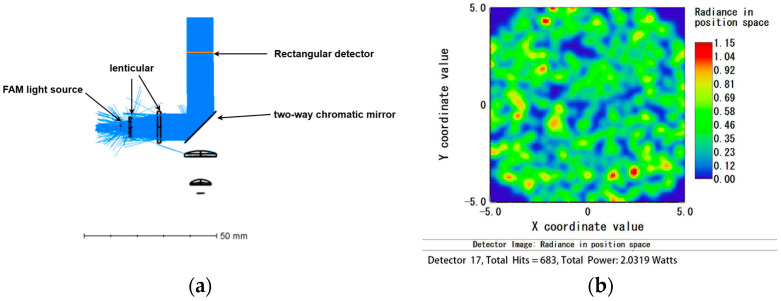
FAM light simulation diagram: (**a**) optical path simulation when FAM light is working alone; (**b**) detector receiving diagram when FAM light is working alone.

The optical path simulation results indicate that the minimum light source efficiency when a single light source is operating is 67.3%, demonstrating a high utilization rate of the light source, which meets the design requirements. The results are shown in Figure 7 and Figure 8.

### 3.2. Experimental Results and Discussion

#### 3.2.1. The Objective of This Study Is to Investigate the Relationship between Nucleic Acid Concentration and the Intensity of the RT-LAMP Signal

**Figure 9:**
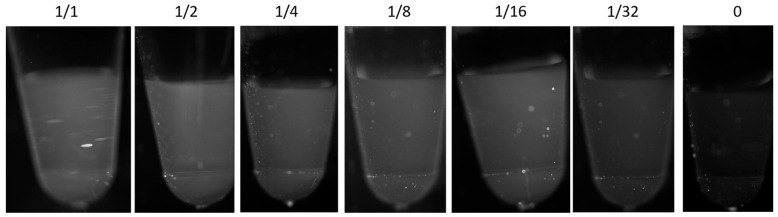
A plot of nucleic acid concentration versus RT-LAMP fluorescence signal intensity.

Positive results from the novel coronavirus 2019-nCoV nucleic acid test kit were diluted multiple times to obtain dilutions of 1/1, 1/2, 1/4, 1/8, 1/16, and 1/32 as positive samples (the type of positive samples is in vitro transcribed RAN containing the N gene with the ORF1ab target gene fragment and the total RNA of the human organism), and 0 was the negative control (the type of negative QC is nuclease-free ultrapure water). The reaction system for the RT-Lamp was 2 × RT-LAMP Premix Buffer II 12.5 μL, 10 × Primers 2.5 μL, BST2.0 HS (8 U/μL) 1 μL, Neoscript RTase (200 U/μL, reverse transcriptase) 0.5 μL, 50 × Eva Green 5 μL, template 5 μL, and ddH_2_O 3.25 μL. The reaction is incubated at 60 °C–65 °C for 60 min and inactivated at 95 °C for 2 min. When a 32-fold dilution of the positive quality control (QC) sample was used as the test sample, the fluorescence intensity of the sample was found to be similar to that of the negative QC. Therefore, in this study, the fluorescence intensity of the 32-fold dilution of the positive QC sample was used as the threshold for distinguishing between positive and negative samples. Samples with fluorescence intensities above the threshold were classified as positive, while samples with intensities below the threshold were classified as negative. This is illustrated in Figure 9.

#### 3.2.2. Repeatable Experiments

Using a pipette, 100 µL of water was added to channels 1 to 4 of the EP tubes. The FAM light detector signal AD values were read 14 times and recorded in Table 2. The data show that the coefficient of variation (CV) values for all four channels are relatively small, with the maximum CV value being 0.09%, indicating good repeatability.

#### 3.2.3. Linear Experiment

The FAM and HEX fluorescent dyes were serially diluted according to Table 3. An amount of 100 µL of the dye solution was added to channel 1, and the corresponding detector AD readings were recorded as shown in Table 3.

**Figure 10:**
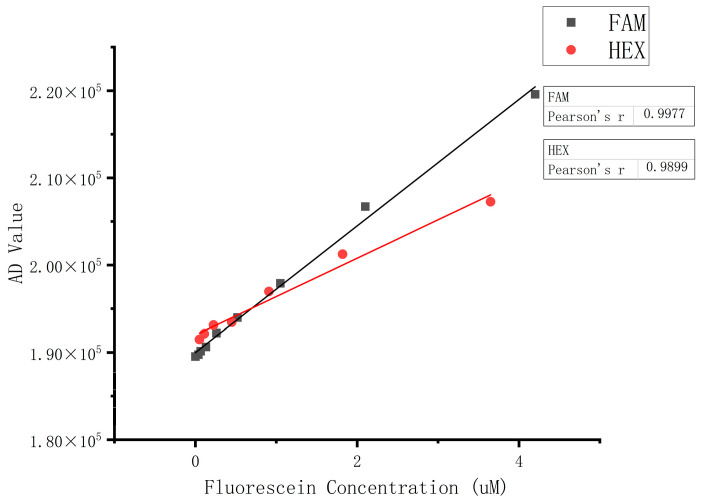
FAM and HEX linear maps.

The FAM and HEX data from Table 3 were subjected to linear fitting, with the fluorescence dye concentration as the x-axis and the detector AD readings as the y-axis. The resulting linear correlation coefficients were R^2^ = 0.9977 for FAM and R^2^ = 0.9899 for HEX, indicating good linearity. Additionally, the slope of the FAM fitting curve is relatively larger, suggesting better resolution. The results are illustrated in Figure 10.

#### 3.2.4. Inter-Channel Interference Experiment

According to the dye concentrations shown in Table 3, 100 µL of dye solution was added to channels 1, 2, and 3, while 100 µL of water was added to channel 4. The AD values of the FAM detection end for the four channels at the corresponding concentrations were recorded as shown in Table 4.

**Figure 11:**
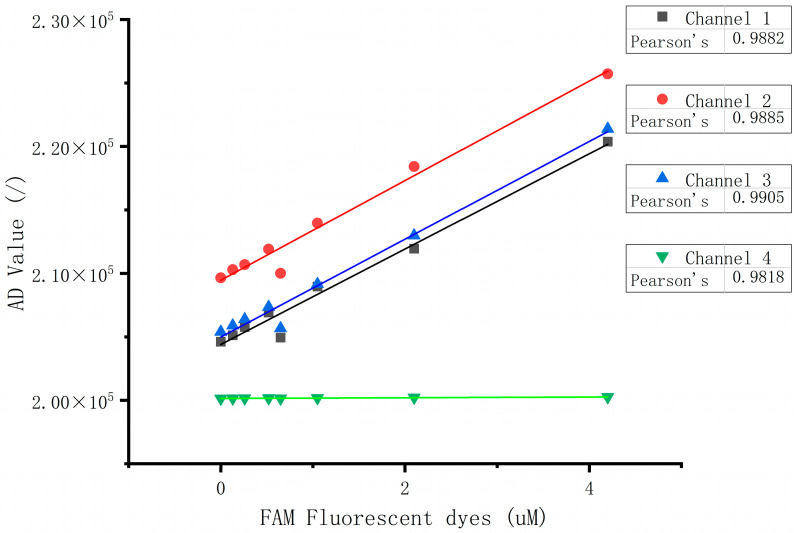
Inter-channel interference map.

The data from Table 4 were plotted with dye concentration as the x-axis and AD values as the y-axis for linear fitting, as shown in Figure 11. The figure demonstrates that channels 1 to 3 exhibit good linear correlation. The detection results for channel 4 show a slight linear increase with the increasing fluorescence concentration in channel 3, indicating good cross-channel interference resistance.

#### 3.2.5. Temperature Performance Test

The benchtop pyrometer probe was placed into channel 3 of the incubator after applying a uniform coating of thermally conductive silicone grease, ensuring that the probe was in close contact with the incubator. The temperature of the incubator was set to 60.5 °C using the device screen (the temperature of the sample solution is usually lower than that of the incubator, so temperature compensation was performed manually, adjusting by 0.5 °C). As shown by the pyrometer, the incubator temperature was stable at 60.641 ± 0.062 °C, with a temperature accuracy of less than 0.2 °C and no obvious temperature overfilling during the warming process. The temperature performance is good and meets the design requirements.

#### 3.2.6. Control Experiment

Using the 2019-nCoV nucleic acid detection kit (Mindray Bio-Medical Electronics, Shenzhen, China), 96 test samples were prepared with positive control samples. These samples were compared with results from a real-time quantitative PCR instrument (Rion Biologicals, Shanghai, China). The positive concordance rate was 100%, and the negative concordance rate was 95.0%. The concordance rate results are shown in Table 5.

### 3.3. Discussion

The most commonly utilized nucleic acid detection devices on the market are digital PCR, real-time fluorescence quantitative PCR, and colloidal gold. Digital PCR is the most sensitive of the techniques, typically achieving a sensitivity of 5~10 copies, and is therefore well-suited to accurately measuring sample concentration. Given the high cost of the instrument and the expense of a single test, it is typically employed in laboratory settings and other similar environments. Real-time fluorescence quantitative PCR is the most prevalent method, regarded as the “gold standard” of nucleic acid detection, primarily utilized in hospitals and disease prevention and control departments. In comparison to digital PCR, real-time fluorescence quantitative PCR, colloidal gold, and other equipment, this device exhibits several advantages, including a compact design, a relatively low cost, a high level of sensitivity, a short reaction time, and so forth. It is therefore suitable for use in a variety of settings, including community hospitals and primary disease prevention screening. For further details, please refer to Table 6.

## 4. Conclusions

A novel point-of-care testing (POCT) device for detecting SARS-CoV-2 based on the RT-LAMP method was designed. The overall design and temperature control schemes of the device were briefly introduced, and the optical path system and incubation chamber heating system were simulated. The simulation results met the design requirements. Additionally, validation experiments were conducted using FAM dye, HEX dye, and a benchtop thermometer to assess the device’s repeatability, linearity, inter-channel interference, temperature accuracy, and temperature stability. 

The experimental results showed that the device’s coefficient of variation (CV) for repeatability was 0.09%. The linearity was 0.9977 for the FAM channel and 0.9899 for the HEX channel. The inter-channel interference was negligible, and the temperature accuracy was within 0.2 °C with a stability of ±0.062 °C. When compared with a real-time quantitative PCR instrument, the positive concordance rate was 100%, and the negative concordance rate was 95.0%.

This study established a dual-excitation light POCT method for detecting SARS-CoV-2 using the RT-LAMP method, and elaborated on the device’s design principles, design scheme, simulation analysis, and performance validation. The device features a compact size, low cost, and ease of use, providing preliminary research for the POCT-based detection of the novel coronavirus.

## Figures and Tables

**Table 1:** Laboratory tools, instruments, and reagents list.

Title	Factory	Model Number	Note
FAM fluorescent dye	Biosafety Biologicals, Shanghai, China	YH0011	Ex/Em: 494/517 nm
HEX fluorescent dye	Biosafety Biologicals, Shanghai, China	YH0010	Ex/Em: 527/558 nm
Benchtop thermometer	XIATECH, Xi’an, China	T1010	/
Pipette	Eppendorf, Hamburg, Germany	/	20 μL, 100 μL
8 rows of EP pipes	Axygen, Silicon Valley, USA	PCR-0208-C	Transparent, 0.2 mL
Novel coronavirus 2019-nCoV nucleic acid test kit	Matilda Biologicals, Wuhan, China	/	Fluorescent dye method
RT-Lamp premix	Biori, Zhuhai, China	HW206-R01	
Real-time fluorescence PCR instrument	Rion Biologicals, Shanghai, China	Vantage MXTM	/

**Table 2:** Record sheet for repeatability experiments.

Serial Number	Channel 1	Channel 2	Channel 3	Channel 4
1	201,490	210,896	251,250	226,027
2	201,228	210,657	250,955	225,751
3	201,211	210,643	250,837	225,629
4	201,195	210,635	251,258	225,573
5	201,186	210,678	251,148	225,544
6	201,176	210,628	251,240	225,522
7	201,175	210,514	251,254	225,515
8	201,170	210,434	251,174	225,462
9	201,164	210,569	251,071	225,403
10	201,162	210,639	250,954	225,390
11	201,164	210,559	250,984	225,365
12	201,164	210,757	250,787	225,336
13	201,167	210,641	250,756	225,317
14	201,172	210,636	251,180	225,298
Average value	201,201.71	210,634.71	251,060.57	225,509.43
Standard deviation	85.25	107.66	180.88	197.38
Coefficient of variation (CV)	0.04%	0.05%	0.07%	0.09%

**Table 3:** Linear experiment record sheet.

Serial Number	FAM Fluorescent Dyes (µM)	FAM_AD	HEX Fluorescent Dyes (µM)	HEX_AD
1	0	189,536	0.051	191,486
2	0.0375	189,760	0.112	192,140
3	0.065	190,156	0.225	193,132
4	0.13	190,156	0.45	193,450
5	0.26	192,220	0.91	196,970
6	0.52	193,988	1.82	201,244
7	1.05	197,914	3.65	207,252
8	2.1	206,710	—	—
9	4.2	219,582	—	—

**Table 4:** Inter-channel interference experiment record sheet.

FAM Fluorescent Dyes (µM)	Channel 1 AD	Channel 2 AD	Channel 3 AD	Channel 4 AD
0	204,624	209,653	205,395	200,143
0.65	204,942	210,010	205,670	200,152
0.13	205,120	210,298	205,883	200,153
0.26	205,780	210,695	206,381	200,157
0.52	206,917	211,906	207,349	200,169
1.05	208,968	213,963	209,155	200,183
2.1	211,954	218,416	212,978	200,217
4.2	220,383	225,724	221,382	200,262

**Table 5:** Record sheet of results of controlled experiments.

This Device	qPCR		Consider
	Positives	Negatives	
Positives	76	1	77
Negatives	0	19	19
	76	20	96

**Table 6:** Comparison table with common testing instruments on the market.

	This Device	Digital PCR	Real-Time Fluorescence Quantitative PCR	Colloidal Gold (Chemistry)
Sample throughput	4	8~32	96	1
Sensitivity	20~50 copies	5~10 copies	10~20 copies	80~100 copies
Price	USD 150–300	USD 30,000–80,000	USD 10,000–15,000	USD 150–300
Dominance	Small size and low price. Relatively high sensitivity, reaction time is usually 40 min to 60 min.	High sensitivity for accurate quantitative measurements. The reaction time is usually 90 min to 120 min.	High sample throughput and high sensitivity. The reaction time is usually 90 min to 120 min. Considered the “gold standard” for nucleic acid testing.	Small size and low instrument price.

## Data Availability

The data that support the findings of this study are available from the corresponding author upon reasonable request.
